# Association between Expression Quantitative Trait Loci and Metabolic Traits in Two Korean Populations

**DOI:** 10.1371/journal.pone.0114128

**Published:** 2014-12-10

**Authors:** Kyung-Won Hong, Seok Won Jeong, Myungguen Chung, Seong Beom Cho

**Affiliations:** 1 Division of Biomedical Informatics, Center for Genome Science, National Institute of Health, KCDC, Cheongwon-gun, Korea; 2 Division of Molecular and Life Science, Hanyang University, Seoul, Korea; Vanderbilt University Medical Center, United States of America

## Abstract

Most genome-wide association studies consider genes that are located closest to single nucleotide polymorphisms (SNPs) that are highly significant for those studies. However, the significance of the associations between SNPs and candidate genes has not been fully determined. An alternative approach that used SNPs in expression quantitative trait loci (eQTL) was reported previously for Crohn’s disease; it was shown that eQTL-based preselection for follow-up studies was a useful approach for identifying risk loci from the results of moderately sized GWAS. In this study, we propose an approach that uses eQTL SNPs to support the functional relationships between an SNP and a candidate gene in a genome-wide association study. The genome-wide SNP genotypes and 10 biochemical measures (fasting glucose levels, BUN, serum albumin levels, AST, ALT, gamma GTP, total cholesterol, HDL cholesterol, triglycerides, and LDL cholesterol) were obtained from the Korean Association Resource (KARE) consortium. The eQTL SNPs were isolated from the SNP dataset based on the RegulomeDB eQTL-SNP data from the ENCODE projects and two recent eQTL reports. A total of 25,658 eQTL SNPs were tested for their association with the 10 metabolic traits in 2 Korean populations (Ansung and Ansan). The proportion of phenotypic variance explained by eQTL and non-eQTL SNPs showed that eQTL SNPs were more likely to be associated with the metabolic traits genetically compared with non-eQTL SNPs. Finally, via a meta-analysis of the two Korean populations, we identified 14 eQTL SNPs that were significantly associated with metabolic traits. These results suggest that our approach can be expanded to other genome-wide association studies.

## Introduction

Recently, large-scale genome-wide association studies (GWAS) that comprised several thousands of samples have reported many novel findings in various diseases and disease-related phenotypes [1]. These findings have been enlightening the path to the identification of disease mechanisms and biomarkers.

Among the human phenotypes, metabolic traits are frequently studied in different populations [2]. In the Korean population, common metabolic traits, such as glucose, cholesterol, and bilirubin levels, have been studied via conventional GWAS [3], [4]. However, little was explained by heritability [3]. This phenomenon, which is termed the missing heritability problem, is hard to resolve by conventional GWAS. It was suggested that the missing heritability might come from the stringent multiple testing correction of GWAS analyses [5]. This multiple testing correction is necessary to exclude false-positive loci, but simultaneously may discard many true-positive loci [6]. In other studies, it was shown that reducing the number of tests is advantageous for GWAS. In that research, the categorization of the genome-wide SNPs into functional categories provided the opportunity to reduce multiple testing and to identify functional variants [7], [8].

In addition to the missing heritability, it should be considered that most of the significant single-nucleotide polymorphisms (SNPs) used in these GWAS lay in intergenic and intron regions and had little association with changes in the protein-coding sequences of genes [1]. Thus, these SNPs likely regulate gene activity at the transcript level directly, or cooperate with other DNA variations that mediate this type of regulation. Based on these facts, expression quantitative loci (eQTL) are being actively studied for elucidating the relationship between changes in genotype and expression dynamics, which will promote the understanding of the results of GWAS [9]–[15].

eQTL information provides insights into the regulation of transcription and aids in the interpretation of genome-wide association studies [9]. In cases in which the allelic changes of a SNP are significantly correlated with the expression of a gene, the SNP is defined as an eQTL-SNP. Using this information, researchers try to identify trait-associated SNPs that would be otherwise hard to find. For example, Fransen and colleagues reported a GWAS for Crohn’s disease using eQTL-SNP information. Those authors selected eQTL SNPs among the GWAS results for Crohn’s disease, and performed follow-up replication studies [6]. They showed that the eQTL-based preselection for follow-up studies was a useful approach for identifying risk loci from the results of a moderately sized GWAS.

Here, we reanalyzed genome-wide associations between metabolic traits and SNPs using eQTL information. The main goal of this research was to explore metabolic trait-associated variants using an eQTL-based filtering strategy. The major eQTL SNPs used in this study were obtained from the RegulomeDB, and the other eQTL SNPs were obtained from recent reports of liver tissues [13] and from lymphoblastoid cell lines [14]. We collected the genotypes of the eQTL SNPs from the Korean Association Resource (KARE) [3], [16] and examined their association with 10 metabolic traits in two independent Korean cohorts (Ansan and Ansung).

## Materials and Methods

### Study subjects

The study subjects comprised two population-based cohorts, Ansung and Ansan, which have been examined as part of the Korean Genome and Epidemiology Study (KoGES). The phenotype of the cohort has been described [17]. Briefly, the subjects came from Ansung and Ansan in KyungGi-Do province, near Seoul, Korea. Written informed consent was obtained from all participants, and this research project was approved by the institutional review board of KNIH. A total of 10,038 individuals were recruited for the cohorts, and 8,842 individuals of the KoGES were analyzed by the Korean Association Resource (KARE) consortium to understand their genome-wide association with the surveyed or measured phenotypes [16].

Subjects with genotype accuracies below 98% and high missing genotype call rates (≥5%), high heterozygosity (>30%), or inconsistency in sex were excluded from subsequent analyses. Individuals who had a tumor were excluded, as were related individuals whose estimated identity-by-state (IBS) values were high (>0.80). Based on these criteria, 8,842 samples were selected; these quality-control steps were described in a previous GWAS [16].

We obtained the following information from the cohorts (Ansung and Ansan cohorts): sex; age; past disease history; anthropometric measurements, such as weight and height; and biochemical measurements, such as fasting glucose (GLU0), serum albumin (ALB), blood urea nitrogen (BUN), aspartate aminotransferase (AST), alanine aminotransferase (ALT), gamma glutamyl-transpeptidase (GGT), total cholesterol (Tchol), high-density lipoprotein cholesterol (HDLC), and triglyceride (TG) levels. Low-density lipoprotein cholesterol (LDLC) was calculated as per the Friedewald formula: LDL = Tchol – HDLC – (TG/5) (all concentrations in mg/dL). The exclusion criteria of each metabolic trait are described in Table 1.

**Table 1:** Clinical characteristics of the Ansung and Ansan cohorts and the exclusion criteria for each biochemical trait.

	Ansung	Ansan	p-value	Exclusion criteria
***Categorical Variables***			Chi-square test p-value	
n (number of individuals)	4205	4637		
Sex ratio (Male/Female)	0.76 (1809/2396)	1.05 (2374/2263)	<0.001	
***Continuous Variables (mean +– standard deviation)***			t-test p-value	
Age (years)	55.67±8.74	49.08±7.86	<0.001	
Body weight (kg)	61.33±9.91	64.67±10.04	<0.001	
Body mass index (BMI, kg/m^2^)	24.46±3.29	24.72±2.96	<0.001	
Fasting glucose (GLU0, mg/dl)	83.42±9.70	84.25±9.89	<0.001	>126 mg/dl, and T2DM history
Albumin (ALB, g/dl)	4.16±0.25	4.33±0.33	<0.001	<3.8, >5.1 g/dl
Blood urea nitrogen (BUN, mg/dl)	13.70±3.00	13.73±2.87	0.592	<6, >20 mg/dl
Gamma glutamyl-transpeptidase(GGT, IU/L)	18.29±9.42	20.07±10.07	<0.001	<8, >46 unit
Aspartate aminotransferase(AST, IU/L)	26.26±5.32	26.26±5.50	0.977	<5, >40 unit
Alanin transaminase(ALT, IU/L)	23.76±9.12	24.74±10.04	<0.001	<7, >56 unit
High density lipoproteincholesterol (HDLC, mg/dl)	49.83±8.76	49.52±8.36	0.203	<40 mg/dl, and history of hyperlipidemia
Low density lipoproteincholesterol (LDLC, mg/dl)	104.28±27.42	113.15±25.03	<0.001	>160 mg/dl, and history of hyperlipidemia
Total cholesterol (Tchol, mg/dl)	178.60±29.15	187.18±27.78	<0.001	>240 mg/dl, and history of hyperlipidemia
Triglyceride (TG, mg/dl)	119.95±36.16	119.73±37.09	0.820	>200 mg/dl, and history of hyperlipidemia

### Genotyping

The genotype data were obtained from KARE, which used the Affymetrix Genome-wide Human SNP array 5.0 and imputed SNPs. The genotype quality-control criteria were as reported in a previous GWAS [16]. Briefly, the criteria for the inclusion of SNPs were a genotype call rate >0.98, a minor allele frequency >0.01, and Hardy–Weinberg equilibrium (HWE) (*p*>1×10^–6^). Ultimately, 352,228 SNPs passed the quality-control process. SNP imputation was performed with IMPUTE [18] using the JPT and CHB sets of HapMap Phase 2 as the reference. After removing SNPs with a MAF <0.01 and a SNP missing rate >0.05, we combined the genotyped SNPs and imputed SNPs; a total of 1.8 million SNPs were used in the subsequent study.

Population stratification was tested by a principal component analysis using the EIGENSOFT software [19]. To prevent overrepresentation of regions with more redundant SNPs, we used the indep-pairwise command in PLINK [20] to reduce linkage disequilibrium between the remaining variants by eliminating any SNP that had a pairwise *r* ^2^>0.3 with any other SNP in a 1500 bp window (step size, 150 bp). This reduced the original dataset to 93,877 SNPs; subsequently, we used smartpca [19].

### eQTL-SNP selection

Most of the eQTL-SNP resources that were used in this analysis are available in online databases, such as RegulomeDB (http://regulome.stanford.edu/), including several published resources for various cell types/tissues, such as monocytes [9], human brain [10], lymphoblastoid cell lines [11], [12], and human liver [13]. The RegulomeDB is one of the most comprehensive eQTL-SNP databases, because it contains rich information about the products of the ENCODE project, such as transcription factor binding sites, chromatin structure, histone modification, and eQTL. The RegulomeDB classifies the human SNPs into seven categories [21], of which Category 1 includes eQTL SNPs with other information about the regulatory elements, such as transcription factor binding, chromatin structure, and histone modification. A total of 39,332 eQTL SNPs were downloaded from the RegulomeDB, Category 1. In addition, we also included two recent eQTL reports based on liver (1,078 SNPs) or lymphoblastoid (907 SNPs) tissues [14], [15]. Finally, a total of 41,317 eQTL SNPs were used to extract the same SNPs from our genotype dataset, and we extracted the 25,658 eQTL SNPs from the KARE SNPs using PLINK [20] software.

### Estimation of the genetic variance that is explained by eQTL or non-eQTL SNPs

The non-eQTL SNPs were defined as the SNPs that excluded the eQTL SNPs and were in perfect linkage disequilibrium with the eQTL SNPs (*r* ^2^ = 1.0 and D′ = 1.0). The genetic variances were computed via GCTA v1.24 [22], which is a tool for estimating the proportion of phenotypic variance that is explained by genome-wide SNPs for complex traits [23]. First, we estimated the pairwise genetic relationship using the make-grm option. Next, we estimated the proportion of phenotypic variance that was explained by the eQTL and non-eQTL SNPs, respectively, based on the restricted maximum likelihood (REML) [23].

### Statistical analyses

The effect of a genotype was analyzed by linear regression. We calculated the effect size (beta) and standard error (SE) of minor alleles on metabolic traits for each Ansung and Ansan subject. All analyses were adjusted for age, sex, body mass index (BMI), and principal component (PC) 1 and PC2. PLINK v 1.07 [20] was used for all statistical tests. All tests were performed based on the additive model, and we combined the Ansung and Ansan results by an inverse-variance meta-analysis under the assumption of fixed effects using Cochran’s Q test, to assess between-study heterogeneity [24]. In this study, we applied false discovery rates and Bonferroni correction *p*-values to determine significant associations.

## Results

### Dataset characteristics

The clinical characteristics and sample exclusion criteria are described in Table 1. The KARE subjects consisted of two Korean populations (Ansung and Ansan), which we used as the replication set of genetic associations based on their differing clinical characteristics. However, the population stratification analysis showed similar genetic structures (S1 Figure), indicating that they constitute a replication set that can be used to identify consistent genetic effects, despite the differences in demographics and environments.

### The proportion of phenotypic variance that was explained by eQTL and non-eQTL SNPs

Based on the RegulomeDB and previous reports, we isolated 25,658 eQTL SNPs from the KARE genotype dataset. The proportion of phenotypic variance that was explained by eQTL and non-eQTL SNPs for complex traits is described in Table 2. The non-eQTL sites examined were approximately 66 times more examined SNPs than the eQTL sites; however, compared with those explained by non-eQTL SNPs, the proportions of phenotypic variance that were explained by eQTL SNPs were larger for GLU0 (1.9 in eQTL vs 0.2 in non-eQTL SNPs) and TG (1.3 in eQTL vs 0.1 in non-eQTL SNPs), and similar for BUN (3.4 in eQTL vs 4.0 in non-eQTL SNPs). Moreover, the proportions of phenotypic variance that were explained by eQTL SNPs were relatively large for the remaining phenotypes, with the exception of ALB.

**Table 2:** Estimated genetic variance explained by eQTL SNPs and non-eQTL SNPs.

	h2	Ref	eQTLs (n = 25,658)			Non-eQTL SNPs (n = 1.7 million)		
			Vg	Vp	Vg/Vp	Vg	Vp	Vg/Vp
GLU0	0.52–0.65	[41]	1.71±0.96	90.13±1.50	1.9±1.1	0.27±3.08	90.72±1.49	0.2±3.4
ALB	0.48–0.82	[44]	0.00±0.00	0.08±0.00	0.0±0.9	0.007±0.003	0.081±0.001	9.1±3.5
BUN			0.27±0.10	8.02±0.13	3.4±1.2	0.32±0.28	8.02±0.13	4.0±3.4
GGT	30	[45]	0.46±0.84	71.69±1.28	0.6±1.2	4.87±2.87	71.69±1.28	6.8±4.0
AST	43	[45]	0.42±0.29	27.24±0.46	1.5±1.0	0.83±0.96	27.24±0.46	3.1±3.5
ALT	40	[45]	1.55±0.94	85.15±1.44	1.8±1.1	7.54±3.07	85.15±1.46	8.9±3.5
HDLC	0.68–0.86	[41]	1.04±1.08	70.52±1.47	1.5±1.5	4.19±3.88	70.52±1.47	5.9±5.5
LDLC	0.37	[42]	26.23±9.22	673.72±12.39	3.9±1.4	99.13±29.70	673.80±12.39	15.7±4.4
Tchol	0.26	[43]	19.19±9.89	781.03±14.19	2.5±1.3	113.77±33.40	781.06±14.20	14.6±4.2
TG	0.39–0.53	[41]	16.17±16.48	1250.96±24.29	1.3±1.3	1.60±57.07	1250.98±24.28	0.1±4.6

Note. H2: previously reported heritability of the trait, Vg: estimated genetic variance, Vp: estimated phenotypic variance, Vg/Vp: percent of estimated genetic variance explained by SNPs for each trait.

### Association study of eQTL-related SNPs

These eQTL SNPs were examined with regard to their association with metabolic traits in the Ansung and Ansan cohorts. Ultimately, we selected 509 eQTL SNPs that had the same effect and *p*-values<0.05 in both cohorts, and combined the results obtained for each cohort via a meta-analysis. All association results and meta-analysis results for the 509 SNPs are described in S1 Table.

Among the 509 SNPs analyzed, we identified significant associations using adjusted *p*-values<0.05 for FDR. Twenty-six SNPs for GLU0, eleven SNPs for GGT, two SNPs for Tchol, thirty-five SNPs for LDLC, and two SNPs for TG met our criteria (S1 Table, characters highlighted in bold). Because many associated SNPs were located in the same LD blocks, we selected the most significant SNP in each significant LD block (summarized in Table 3). We then used these SNPs for further annotation and compared them with the previous conventional GWAS results (http://www.genome.gov/gwasstudies). Further information for the significant eQTL positions is described in Table 4, including the genes, cell types, and ENCODE regulatory elements in metabolic-trait-related cell types derived from the blood, liver, or pancreas. No significant association was detected regarding the results of ALB, BUN, AST, ALT, and HDLC.

**Table 3:** Significantly associated SNPs in the Ansung and Ansan cohorts and meta-analysis results.

CHR	SNP	BP	A1	MAF	Ansan			Ansung			Meta-analysis					
					Beta	SE	P-value	Beta	SE	P-value	Beta	P-value	Cochran's Q	Heterogeneity	FDR	BONF
*Fasting glucose (GLU0)*																
11	rs1535	61354548	G	0.31	−0.997	0.226	1.0E-05	-0.871	0.261	8.4E-04	−0.943	3.3E-08	0.71	0.00	4.0E-04	8.4E-04
6	rs463302	33352694	C	0.09	1.254	0.374	8.1E-04	1.453	0.401	2.9E-04	1.347	8.5E-07	0.72	0.00	1.3E-03	2.2E-02
6	rs3002007	56726719	T	0.01	2.551	0.911	5.1E-03	3.403	1.007	7.4E-04	2.934	1.4E-05	0.53	0.00	1.6E-02	0.358
1	rs1431985	212214869	A	0.45	−0.669	0.214	1.8E-03	-0.620	0.237	9.1E-03	-0.647	4.7E-05	0.88	0.00	4.7E-02	1.000
*Gamma-glutamyl transpeptidase (GGT)*																
12	rs2251468	119889509	C	0.49	−1.014	0.213	2.1E-06	-0.574	0.232	1.3E-02	−0.812	2.2E-07	0.16	48.93	1.3E-03	5.7E-03
12	rs11065774	109839709	A	0.17	−0.943	0.269	4.6E-04	−1.082	0.285	1.5E-04	−1.009	2.5E-07	0.72	0.00	1.3E-03	6.3E-03
7	rs13233571	72609167	T	0.10	−1.201	0.351	6.3E-04	−0.987	0.360	6.2E-03	−1.096	1.3E-05	0.67	0.00	4.0E-02	0.327
*Total cholesterol (Tchol)*																
2	rs780092	27596658	G	0.33	−2.416	0.720	7.9E-04	−3.079	0.806	1.4E-04	−2.710	4.4E-07	0.54	0.00	9.8E-03	1.1E-02
5	rs4604177	74844636	C	0.47	−2.724	0.677	5.8E-05	−2.203	0.755	3.6E-03	−2.492	7.7E-07	0.61	0.00	9.8E-03	2.0E-02
*Low density lipoprotein cholesterol (LDLC)*																
12	rs11065774	109839709	A	0.17	1.909	0.822	2.0E-02	3.647	0.946	1.2E-04	2.657	1.8E-05	0.17	48.03	4.6E-02	0.474
18	rs9966367	59728271	G	0.37	2.096	0.640	1.1E-03	1.900	0.742	1.0E-02	2.012	3.3E-05	0.84	0.00	4.6E-02	0.844
5	rs4604177	74844636	C	0.47	−2.019	0.623	1.2E-03	−1.711	0.722	1.8E-02	−1.888	6.3E-05	0.75	0.00	4.6E-02	1.000
*Triglyceride (TG)*																
8	rs12679834	19864713	C	0.12	−6.528	1.457	7.8E-06	−5.804	1.528	1.5E-04	−6.183	4.5E-09	0.73	0.00	6.0E-05	1.2E-04
11	rs651821	116167789	C	0.27	5.592	1.151	1.2E-06	4.293	1.279	8.0E-04	5.011	4.7E-09	0.45	0.00	6.0E-05	1.2E-04

Note. CHR: chromosome, SNP: single-nucleotide polymorphism, BP: base position based on the human genome (NCBI36/hg18), A1: minor allele, MAF: minor allele frequency, Beta: effect size, SE: standard error, FDR: adjusted p-value by false discovery rate, BONF: adjusted p-value by bonferroni correction.

**Table 4:** In silico annotation of eQTLs.

CHR	BP	SNP	lead SNP Position	eQTL gene symbol	Gene Description	Distancefrom SNP (kb)	eQTL Cell type	TF	DHS	Histon Modification
***Fasting glucose (GLU0)***										
11	61354548	rs1535	Intron ofFADS2	FADS1	Fatty acid desaturase 1	26,5	Lymphoblastoid	O	O	O
				NXF1	Nuclear RNAexport factor 1	968.0				
6	33352694	rs463302	3′ flanking ofB3GALT4	B3GALT4	UDP-Gal:betaGlcNAcbeta 1,3-galactosyltransferase,polypeptide 4	0.2	Cerebellum	O	O	O
6	56726719	rs3002007	Intron of DST	RAB23	RAB23, member RASoncogene family	360.3	Monocytes	O	O	O
1	212214869	rs1431985	upstream ofAK092251	TRAF5	TNF receptor-associatedfactor 5	666.5	Liver	O		O
***Gamma-glutamyl transpeptidase (GGT)***										
12	119889509	rs2251468	downstream ofHNF1A-AS1	C12orf43	Chromosome 12 openreading frame 43	37.0	Monocytes		O	O
12	109839709	rs11065774	Intron ofMYL2	MYL2	Myosin lightchain 2	-	Lymphoblastoid	O	O	O
7	72609167	rs13233571	Intron ofBCL7B	TBL2	transducin(beta)-like 2	383.8	Lymphoblastoid	O	O	O
***Total cholesterol (Tchol)***										
2	27596658	rs780092	Intron ofGCKR	XAB1	XPA bindingprotein 1, GTPase	277.1	Lymphoblastoid			O
5	74844636	rs4604177	Intron ofPOLK	FAM169A	Family with sequencesimilarity 169	617.1	Lymphoblastoid		O	O
***Low density lipoprotein cholesterol (LDLC)***										
12	109839709	rs11065774	Intron ofMYL2	MYL2	Myosin lightchain 2	-	Lymphoblastoid	O	O	O
18	59728271	rs9966367	Intron ofSERPINB10	SERPINB10	serpin peptidase inhibitor,clade B (ovalbumin), member 10	44.1	Lymphoblastoid	O	O	O
5	74844636	rs4604177	Intron ofPOLK	FAM169A	Family with sequencesimilarity 169	617.1	Lymphoblastoid		O	O
***Triglyceride (TG)***										
8	19864713	rs12679834	Intron ofLPL	LPL	Lipoproteinlipase	-	Monocytes		O	O
11	116167789	rs651821	5′ UTR ofAPOA5	TAGLN	Transgelin	412.9	Monocytes	O	O	O

Note. CHR: chromosome, BP: base position based on the human genome (NCBI36/hg18), SNP: single-nucleotide polymorphism, eQTL: expression quantitative trait loci, TF: Transcription factor binding in liver or pancreas cells, DHS: DNase1 Hypersensitive site in liver or pancreas cells.

### Significantly associated eQTL SNPs

Table 3 shows that two SNPs (rs1535 and rs463302) for FPG, two SNPs (rs2251468 and rs11065774) for GGT, two SNPs (rs780092 and rs4604177) for Tchol, and two SNPs (rs12679834 and rs651821) for TG were significantly associated and passed the Bonferroni correction. The *p*-values of two SNPs (rs3002007 and rs1431985) for FPG, one SNP (rs13233571) for GGT, and three SNPs (rs11065774, rs9966367, and rs4604177) for TG did not pass the Bonferroni correction, but passed the FDR correction.

In this study, we focused on significantly associated SNPs. SNP rs1535 was a *FADS1* and *NXF1* eQTL-SNP hot spot, and the results of the meta-analysis of the association between the Ansan and Ansung populations were *β* = –0.943 and *p*-value = 3.3×10^–8^. SNP rs463302 was a *B3GALT4* eQTL-SNP hot spot, and the results of the meta-analysis were *β* = 1.347 and *p*-value = 8.5×10^–7^. SNP rs2251468 was a *C12orf43* eQTL-SNP hot spot, and the results of the meta-analysis were *β* = –0.812 and *p*-value = 2.2×10^–7^. SNP rs11065774 was an *MYL2* eQTL-SNP hot spot, and the results of the meta-analysis were *β* = –1.009 and *p*-value = 2.5×10^–7^. SNP rs780092 was a *XAB1* eQTL-SNP hot spot, and the results of the meta-analysis were *β* = –2.710 and *p*-value = 4.4×10^–7^. SNP rs4604177 was a *FAM169A* eQTL-SNP hot spot, and the results of the meta-analysis were *β* = –2.492 and *p*-value = 7.7×10^–7^. SNP rs12679834 was an eQTL-SNP hot spot of *LPL*, and the results of the meta-analysis were *β* = –6.183 and *p*-value = 4.5×10^–9^. Finally, SNP rs651821 was an eQTL-SNP hot spot of *TAGLN*, and the results of the meta-analysis were *β* = 5.011 and *p*-value = 4.7×10^–9^.

## Discussion

In this study, we performed a GWAS of 10 biochemical traits using SNPs that were preselected based on the eQTL-SNP lists of RegulomeDB and two recent eQTL papers. The proportion of phenotypic variance that was explained by eQTL- and non-eQTL-related SNPs showed that the eQTL SNPs were more likely to be associated with the metabolic traits than were the non-eQTL SNPs. We identified 14 eQTL SNPs that were associated with metabolic traits in two Korean populations (Ansung and Ansan), in which the *p*-values met our multiple comparison criteria. The SNPs revealed novel candidate genes for FPG, GGT, Tchol, LDLC, and TG.

SNP rs1535 was reported as being associated with decreased plasma phospholipid levels by an European study [25], and with decreased HDLC by an Indian Asian study [26]. Although *FADS1* has been studied extensively in lipid metabolism, recent genetic association studies showed its association with glucose metabolism [27]. Moreover, NXF1, a novel glucose-regulated protein, is elevated under high-glucose conditions [28]. Therefore, the expression of both the *FADS1* and *NXF1* genes is influenced by the rs1535 genotypes, which might represent a functional element for regulating both glucose and lipid metabolism.

SNP rs463302 has not been reported in association studies; however, our study indicated that rs463302 may contribute to *B3GALT4* expression levels. *B3GALT4* encodes UDP-Gal:betaGlcNAc beta 1,3-galactosyltransferase, polypeptide 4, which is a member of the beta-1,3-galactosyltransferase protein family. Although B3GALT4 mitigates ganglioside activity in neurodegenerative disorders, such as Huntington disease [29], it was also reported that gangliosides are related to the immunological pathophysiology of type 1 diabetes and to insulin resistance in type 2 diabetes [30], [31]. SNP rs463302 lies 200 bp upstream of *B3GALT4*, to which RNA polymerase 2A (POLR2A) binds, based on the ENCODE ChIP-seq data. In addition, the ENCODE DNase-seq and histone modification data indicated that the chromatin around this SNP is open and undergoes histone modifications. Taken together, our results and *in silico* evidence suggest that the rs463302 SNP may be a regulatory factor of *B3GALT4* and a modifying factor of fasting glucose level.

In our study, rs2251468 was significantly associated with GGT. The SNP was recently reported to be associated with plasma homocystein levels [32]. Because homocystein concentration is a risk factor for coronary artery disease [32] and is significantly correlated with GGT [33], this SNP might be a novel candidate marker for coronary artery disease.

Although rs12679834 has not been reported in association studies, a SNP that is in high LD with rs12679834 (rs331, *r* ^2^ = 0.771, D′ = 1.000) has been reported as being associated with plasma lipoprotein concentration [34]. SNP rs12679834 was an eQTL-SNP of *LPL* (lipoprotein lipase), which is a critical enzyme in lipid metabolism that catalyzes the hydrolysis of TGs. Dysfunction of *LPL* induces pathophysiological lipid-related disorders, including hyperlipidemia, dyslipidemia [35], and hypertriglyceridemia [36].

Our study had two limitations. First, although the positions of the eQTL SNPs have several evidences of the regulatory elements from ENCODE results, not all eQTL SNPs were experimented in the cell types or tissues that are directly related to the metabolic traits [37]. Thus, we assumed that the eQTL SNPs also played a similar role in the metabolic-trait-related cell types or tissues. For example, the *B3GALT4* eQTL-SNP (rs463302) was experimented in cerebellum tissue; however, it is located on several ENCODE regulatory elements, such as transcription factor binding, open chromatin, and active histone modification markers, in metabolic-trait-related cell types, such as the liver and pancreas. The other limitation was that we used HapMap 2 imputed SNPs. Recently, many genome-wide association studies used 1000 Genomes Project-based imputation [38]. *Although we could not use 1000 Genomes-based imputation in the current study, we will apply it in future studies.*

Previous GWASs of complex traits have primarily examined DNA sequence variants (DSVs) between individuals that contribute to the susceptibility to a disease, clinical outcomes, and response to therapy [9]. However, the mechanisms that govern the expression of a phenotype are embedded not only in the DSVs, but also through their effects on various genomic components that regulate gene expression, variants, and posttranslational modifications of the encoded proteins, in conjunction with environmental factors. Thus, a complicated phenotype is the consequence of complex interactions between many genetic and nongenetic factors.

The results of eQTL-SNP GWAS might be novel targets for the design of future experimental studies. As an example, rs12740374 was identified in European and American GWAS for LDLC, and those studies reported the *CELSR2* gene as a candidate gene based on positional proximity [39]. However, further analysis showed that the SNP is an eQTL of *SORT1* [40]. Moreover, our approach using eQTL SNPs provides the possibility of understanding the internal mechanism that underlies the link from SNP to genes to phenotype. For example, in our study, SNP rs1535 was an eQTL of *NXF1*, which is regulated by the blood glucose levels. This result led us to speculate on the following internal link: the modulation of *NXF1* expression by blood glucose levels may be modified by eQTL SNPs.

In conclusion, the relationships between functional eQTL SNPs and 10 biological traits may be helpful for understanding the underlying mechanism that connects genotype and phenotype. Because eQTL SNPs promote the understanding of gene expression and regulation, this approach might help identify biomarkers of metabolic traits.

## Supporting Information

S1 Figure Population stratification of Ansung and Ansan. The PCA analysis by using the Affymetrix 5.0 SNP array were conducted by EIGENSTAT.
(PPTX)

S1 Table The all associated eQTLs in both Ansan and Ansung.
(XLSX)
