# Supplementary material for: Association between Expression Quantitative Trait Loci and Metabolic Traits in Two Korean Populations
Source: PLoS One. 2014 Dec 10;9(12):e114128. doi: 10.1371/journal.pone.0114128 (PMC4262412; doi:10.1371/journal.pone.0114128)
Supplement: S1 Figure — Population stratification of Ansung and Ansan. The PCA analysis by using the Affymetrix 5.0 SNP array were conducted by EIGENSTAT. (PPTX) [file pone.0114128.s001.pptx]

## Slide 1
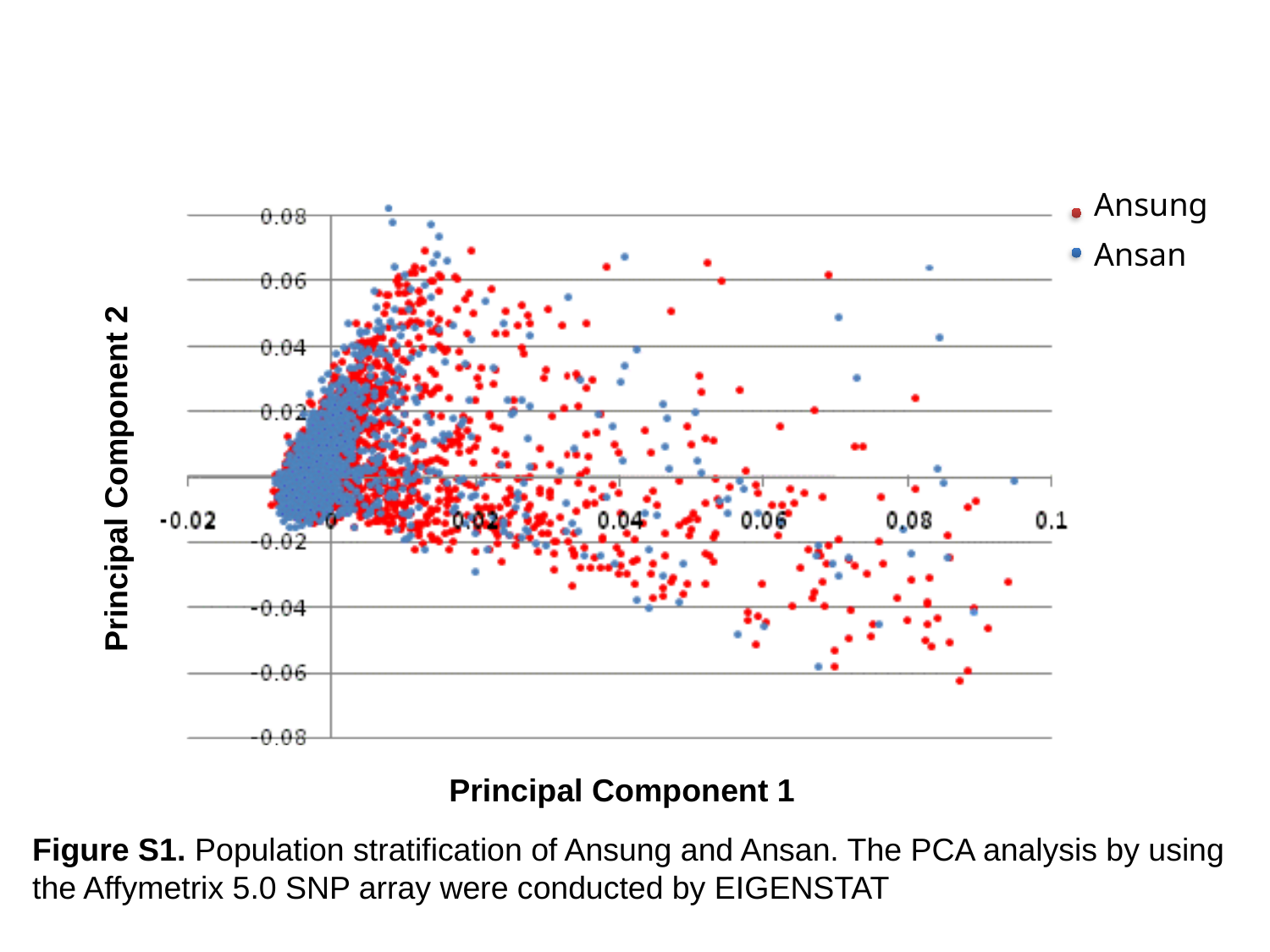

Ansung
Ansan
Principal Component 2
Principal Component 1
Figure S1. Population stratification of Ansung and Ansan. The PCA analysis by using the Affymetrix 5.0 SNP array were conducted by EIGENSTAT
